# Metabolomic Analysis of Methyl Jasmonate-Induced Triterpenoid Production in the Medicinal Herb *Centella asiatica* (L.) Urban

**DOI:** 10.3390/molecules18044267

**Published:** 2013-04-11

**Authors:** Jacinda T. James, Fidele Tugizimana, Paul A. Steenkamp, Ian A. Dubery

**Affiliations:** 1 Department of Biochemistry, University of Johannesburg, Auckland Park 2006, South Africa; 2 Drug Discovery Division, BioSciences, CSIR, Pretoria 0001, South Africa

**Keywords:** *Centella asiatica*, centelloids, triterpenoids, metabolic profiling, metabolomics, LC-MS

## Abstract

*Centella asiatica* is an important source of biologically active pentacyclic triterpenoids. The enhancement of the biosynthesis of the centellosides by manipulation of associated metabolic pathways is receiving much attention. Jasmonates play critical roles in plant metabolism by up-regulating the expression of genes related to secondary metabolites. Here, we investigated the effect of methyl jasmonate (MeJa) in *C. asiatica* through targeted metabolomic profiling of asiaticoside and madecassoside as well as their aglycones, asiatic acid and madecassic acid. Cell suspensions were treated with 0.2 mM MeJa for 2, 4 and 6 days. Liquid chromatography coupled to mass spectrometry (LC-MS) was used to explore induced changes in metabolite profiles, both qualitatively and quantitatively. Principal component analysis (PCA)-derived scores plots revealed clusters of sample replicates for control and treated samples at 2, 4 and 6 days while loading plots aided in identifying signatory biomarkers (asiatic acid and madecassic acid, as well as asiaticoside and madecassoside) that clearly demonstrate the variability between samples. In addition to increased biosynthesis of the targeted centelloids, other differential changes in the intracellular metabolite profiles reflected the response of the *C. asiatica* cells to the MeJa-treatment as a reprogramming of the metabolome.

## 1. Introduction

Plants have the ability to synthesize an enormous variety of secondary metabolites since they need to respond to a continuously changing, and often hostile environment in order to survive and reproduce [1]. Plants that come under attack by pathogens and insects, or are mechanically damaged, produce signal molecules, jasmonic acid (JA) and methyl jasmonate (MeJa), which accumulate in the damaged parts of the plant [2].

Various elicitors have been reported to control metabolic flux between competing metabolic pathways, such as the enhancement of biosynthetic activity of cultured cells by MeJa treatment [3]. Metabolic pathways that originate from squalene, a common precursor for the synthesis of triterpenes (C_30_) and sterols (C_18-29_), form an extensive range of end products [4]. The triterpenoids from *Centella asiatica*, namely madecassoside and asiaticoside, along with their sapogenins madecassic- and asiatic acid, result from the cyclization of 2,3-oxidosqualene by β-amyrin synthase, a specific oxidosqualene cyclase [4,5]. Studies suggest that elicitor action by MeJa affects some steps of terpenoid biosynthesis which leads to increases in triterpenoid saponin levels in aerial parts of the plant. A few jasmonate-responsive genes involved in the biosynthetic pathways in *C. asiatica* have been cloned [6]; these include the genes coding for the enzymes farnesyl diphosphate synthase [7], squalene synthase [8], and oxidosqualene synthase [9]. The jasmonate signalling pathway is connected to other signalling pathways, thus constituting a complex regulatory network. The genes up-regulated by MeJa treatment include those involved in jasmonate biosynthesis, secondary metabolism, cell-wall formation, and those encoding stress-protective and defense proteins [10].

Due to its medicinal properties, there has been an interest to overproduce the centellosides of *C. asiatica* through *in vitro* culture [4,11,12]. The effects of a number of different elicitors, including yeast extract (YE), cadmium chloride, copper chloride and MeJa have been used to enhance asiaticoside production in cultured whole plants [5,13], with only MeJa and YE being able to significantly stimulate asiaticoside production. Different approaches to modify the biosynthetic pathway have also been carried out, given that the last steps of the biosynthetic pathway for triterpenoid saponins are still unknown [14,15], and are likely to involve enzymes that catalyse reactions such as oxidation and glycosylation [16]. The most common strategy is feeding plant cell cultures with commercially available or easily extractable metabolic precursors and substrates [6,17].

Both primary and secondary metabolites comprise the metabolome, with the latter often being species specific and playing a role in the interaction of a cell with its environment [18]. Previously, the analyses of metabolites have focused on small metabolites, but since information about novel compound classes and new metabolic pathways has increased, it has been realized that metabolic pathways do not operate in isolation but are part of extensive networks. 

Metabolomics can contribute significantly towards the investigation of the biology of stress responses in plants by identifying different compounds such as by-products of stress metabolism, stress signal transduction molecules or molecules that are involved in adaptive responses. This is due to improved analytical capabilities, together with newly designed, dedicated statistical, bioinformatics and mining strategies [2]. Metabolomic approaches can thus now provide a more detailed metabolic picture of a living organism under specific conditions [19], and contribute to the understanding of how plants are organized and how metabolism can be both highly flexible and controllable [1].

Altered metabolic profiles of secondary metabolites extracted from MeJa treated *C. asiatica* cell suspensions might be indicative of the potential to manipulate the synthesis of centellosides and other related terpenes. The purpose of this study was to qualitatively and quantitatively analyse the changes in four targeted triterpenoids, (asiatic acid, madecassic acid, asiaticoside and madecassoside) in *C. asiatica* cell suspensions subsequent to exogenous MeJa treatment, by means of chromatographic techniques and multivariate statistical models. To date, a metabolic approach to MeJa-induced secondary metabolism in *C. asiatica* cell suspensions, using metabolomic tools, has previously not yet been described.

## 2. Results and Discussion

Both concentration- and time-studies for MeJa exposure were investigated to optimise experimental conditions that were evaluated by TLC screening of the four centelloids. A concentration of 0.2 mM MeJa was found to be optimal, which is consistent with other studies [20,21,22,23]. After 4 days of 0.2 mM MeJa treatment (a total of 13 days in batch culture) the cells still maintained a viability of >80%.

### 2.1. Partial Characterization and Fractionation of the Plant Extracts by TLC Analysis

TLC analysis of ethanolic extracts of MeJa-treated *C. asiatica* cells indicated an increase in the amount of anisaldehyde/sulphuric acid (AS) reactive bands for the extracts after 4 days of treatment and an increase in the four centellosides was observed (not shown) [24]. In addition, fluorescent compounds not corresponding to any of the four targeted triterpenoids, nor reacting to the AS spray, were detected under UV (366 nm) light, indicating a broader cellular response to MeJa treatment.

### 2.2. Dynamic Changes in the Metabolome of MeJa-treated *C. asiatica* Cells

**Figure 1:**
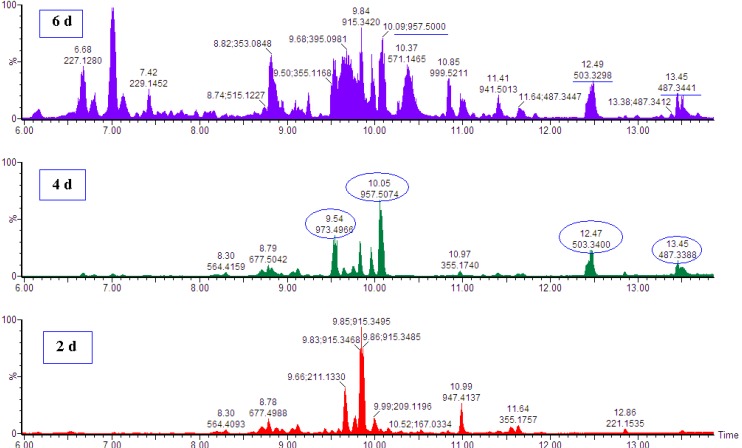
UHPLC-MS (BPI) chromatograms showing treatment-related variations. Ten day old cell suspensions were treated with 0.2 mM MeJa for 2, 4 and 6 days and intracellular metabolites extracted with ethanol. Four ion peaks (*m/z* 973.5, 957.5, 503.3 and 487.3) were more predominantly observed in the 4 days samples than in other conditions (2 and 6 days). These ion peaks were further identified as the targeted triterpenoids/centelloids (Table 1).

**Figure 2:**
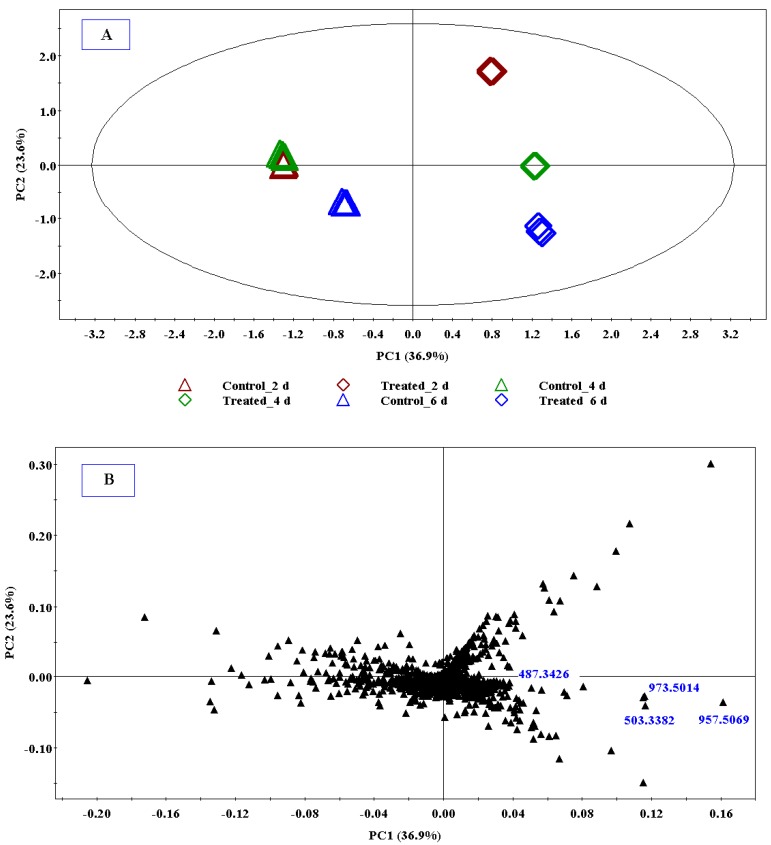
(**A**) PCA scores plot of a representative experiment. A 4-component model explains 87.2% variation. The plot was computed using the first two components (PC1 and PC2) which explain 60.5% of the variation (**B**) PCA loading plot for all time points (2, 4 and 6 days). The mass ions *m/z* = 487.3426, 957.5069, 503.3382 and 973.5014, identified as asiatic acid, asiaticoside, madecassic acid and madecassoside respectively, contribute to the clustering of the samples.

Ultra high performance liquid chromatography coupled to high definition mass spectrometry (UHPLC-HDMS) was used for the analysis. The ESI^+^ mode of MS analysis for the centelloids resulted in decreased sensitivity and produced extensive fragmentation patterns, hence the ESI^−^ mode was used [25,26]. Visual inspection of the base peak intensities (BPI) chromatograms (Figure 1) clearly indicate that 0.2 mM MeJa treatment induced differential metabolic changes in *C. asiatica* cells as exemplified by an increase or decrease in peak intensities and appearance of new ion peaks. The UHPLC-MS data sets were further analysed by multivariate analysis (MVA) to highlight the MeJa-induced metabolic changes (Figure 2).

For metabolomic analysis, bilinear factor models that are most commonly used are principal component analysis (PCA, an unsupervised analysis based only on the explanatory variables) and partial least square discriminant analysis (PLS-DA, a supervised analysis where the user can predefine groups such as time points within the samples) [27,28,29,30]. Here, the data matrix obtained from MarkerLynx XS™ processing was exported into the SIMCA-P 13 software for PCA modelling.

The quality of the PCA models was evaluated based on the diagnostic tools (i) the cumulative modelled variation in matrix X, R^2^X(*cum*) and (ii) the predictive ability parameter, Q^2^(*cum*): the fraction of the total variation of matrix X that can be predicted by the extracted components. A four-component model was computed that explained 87.2% of the total variation in the X matrix [R^2^X(cum) of 0.872] with the goodness of prediction [Q^2^(cum)] of 0.742. For a robust mathematical model with a reliable predictive accuracy, the values of these diagnostic parameters should ideally be close to 1.0 (or above 0.5) with the difference between these less than 0.2.

A scores plot was constructed using the first two components (PC1 and PC2, explaining 60.5% of the variance), showing samples differentially clustered into different groups with minimal variation within groups. The PCA models provided a global and qualitative visual representation of similarity/dissimilarity between and within the samples (on *x*- and *y*-axes respectively), without using class information: all the non-treated samples (controls) are grouped together and significantly separated from the 0.2 mM MeJa-treated samples (Figure 2A). Since the cells were continuously grown in batch culture, even non-treated cell cultures exhibit dynamic metabolite profiles due to their changing micro-environments.

While the PCA scores plot gives visual information about sample variations, the PCA loading or scatter plot explains the variation in scores and illustrates the putative discriminating variables responsible for sample clustering [27,28,29,30]. The PCA loading plot (Figure 2B) shows discriminating mass ions contributing to the sample clustering in the scores plot. The mass ions, *m/z* = 957.5069, 973.5014, 487.3426 and 503.3382, were identified as corresponding to asiaticoside, madecassoside, asiatic acid and madecassic acid respectively (below). 

**Table 1:** Selected, tentatively identified metabolites in *C. asiatica* cells responding to 0.2 mM MeJa treatment. Metabolites are listed according to decreasing variable importance in projection (VIP) scores.

Rt (min)	Observed mass (*m/z*)	Calculated mass (*m/z*)	Ion	Identification	Molecular Formula	Molecular Weight (g/mol)	Correlation with MeJa effect
10.16	533.1980	533.1970	[M+Na−H]^−^	Gibberellin 2-*O*-beta-d-glucoside	C_25_H_34_O_11_	509.53	Negative
10.05	957.5070	957.5059	[M−H]^−^	Asiaticoside *	C_48_H_78_O_19_	959.12	Positive
9.54	973.5010	973.5008	[M−H]^−^	Madecassoside *	C_48_H_78_O_20_	975.12	Positive
12.47	503.3380	503.3373	[M−H]^−^	Madecassic acid *	C_30_H_48_O_6_	504.70	Positive
9.79	207.1380	207.1385	[M−H]^−^	3-Hydroxy-9-apo-delta-caroten-9-one	C_13_H_20_O_2_	208.29	Positive
8.94	323.0920	323.0919	[M+FA+Na−H]^−^	7,4'-Dihydroxy-8-methylflavan	C_16_H_16_O_3_	256.29	Negative
13.50	487.3430	487.3424	[M−H]^−^	Asiatic acid *	C_30_H_48_O_5_	488.70	Positive
9.23	341.1030	341.1025	[M−H]^−^	4',5,6,7-Tetramethoxyflavone	C_19_H_18_O_6_	342.34	Negative
8.83	353.0870	353.0873	[M−H]^−^	Chlorogenate	C_16_H_18_O_9_	354.31	Positive
7.09	227.1280	227.1283	[M+FA-H]^−^	Geranyl formate	C_11_H_18_O_2_	182.26	Positive
9.69	395.0980	395.0970	[M+Na_Na-H]^−^	Feruloylserotonin	C_20_H_20_N_2_O_4_	352.38	Positive
9.52	355.1180	355.1182	[M−H]^−^	Kievitone	C_20_H_20_O_6_	356.37	Negative
14.74	221.1540	221.1542	[M−H]^−^	Rishitin	C_14_H_22_O_2_	222.32	Positive
10.06	993.4860	993.4848	[M+NaCl_HCOONa−H]^−^	Pheophytin a	C_55_H_72_N_4_O_5_	869.18	Positive
9.07	311.0920	311.0909	[M−H]^−^	Baicalein 5,6,7-trimethyl ether	C_18_H_16_O_5_	312.31	Negative
9.87	251.1280	251.1283	[M−H]^−^	Ubiquinol	C_14_H_20_O_4_	252.30	Positive
10.13	251.0700	251.0783	[M−H]^−^	4'-*O*-Methylisoflavone	C_16_H_12_O_3_	252.26	Negative
10.65	531.1500	531.1495	[M−H]^−^	Flavonol 3-*O*-d-xylosylglucoside	C_26_H_28_O_12_	532.49	Positive
9.86	263.1290	263.1279	[M−H]^−^	Abscisate	C_15_H_20_O_4_	264.31	Positive
10.99	265.1440	265.1433	[M+FA−H]^−^	Glutinosone	C_14_H_20_O_2_	220.30	Positive
9.63	293.0820	293.0814	[M−H]^−^	Dehydrocycloguanandin	C_18_H_14_O_4_	294.30	Negative
7.25	345.1340	345.1338	[M−H]^−^	Gibberellin A3/6/29/34-catabolite	C_19_H_22_O_6_	346.37	Negative
8.84	351.0710	351.0716	[M−H]^−^	4-Methylumbelliferone glucuronide	C_16_H_16_O_9_	352.29	Positive
8.98	383.1130	383.1113	[M−H]^−^	Anthocyanin 3'-*O*-beta-d-glucoside	C_21_H_20_O_7_	384.38	Negative
11.69	942.5160	941.5110	[M−H]^−^	Soyasaponin I	C_48_H_78_O_18_	943.12	Positive
14.53	476.2780	476.2777	[M−H]^−^	LysoPE(0:0/18:2(9Z,12Z))	C_23_H_44_NO_7_P	477.57	Positive
8.95	209.1170	209.1145	[M−H]^−^	Jasmonic acid	C_12_H_18_O_3_	210.27	Positive
13.37	485.3280	485.3297	[M−H]^−^	Gypsogenate	C_30_H_46_O_5_	486.68	Positive
11.40	488.3470	488.3435	[M−H]^−^	Bayogenin	C_30_H_48_O_5_	488.70	Positive

* Definitive identification with authentic standards.

**Figure 3:**
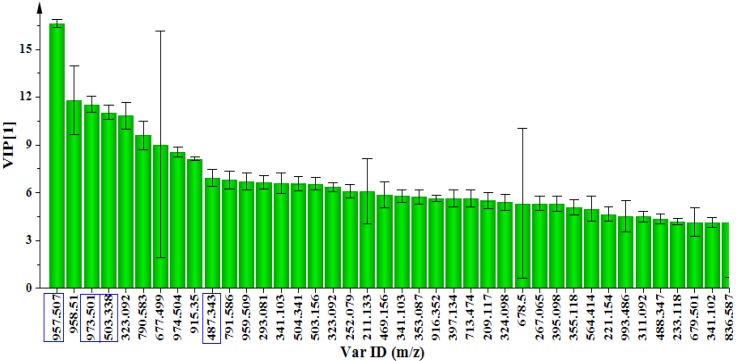
A variable importance in projection (VIP) plot of day 4 samples. The VIP plot indicates that the mass ions *m/z* 487.3426, 503.3382, 957.5069 and 973.5014 were accountable for the significant separation in the model as their VIP scores were significantly greater than 1.0.

The evaluation of the PCA loading plot (Figure 2B) permitted the extraction of statistically and potentially biochemically significant mass ions (metabolites/bio-markers) in the samples. The significance of the selected mass ions in PCA models was also assessed using the variable importance in projection (VIP) plot (Figure 3). The VIP algorithm from SIMCA P+ software is a variable selection method that can enhance the understanding and interpretability of a multivariate model. The VIP plot summarises the importance of the variables and the VIP score is a critically important check on the selection of significant ions/variables in complex metabolomics data sets. The higher the VIP value (exceeding 1.0), the more significant is the ion/variable in the complex analysis in comparing difference between two or more groups [31,32,33,34]. 

The targeted pentacyclic triterpenoids, asiatic acid, madecassic acid, asiaticoside and madecassoside, made highly significant contributions to the model as shown by their VIP >> 1.0 (Figure 3). Thus, this suggests that the MeJa induced a significant increase in the level of the afore-mentioned triterpenoids in the *C. asiatica* cells post-treatment. This observation was confirmed by the quantification of these four compounds and the results are shown in Section 2.3.

Metabolite identification is one of the essential steps in metabolomic analyses since biochemical interpretation of metabolomic data relies on the chemically annotated molecules from spectrometric/spectroscopic signals [30,35]. The selected mass ions from the loading- and VIP-plots were putatively identified using the Taverna workbench (www.taverna.org.uk) [36] for PUTMEDID_LCMS Metabolite ID Workflows [37]. Furthermore, the four targeted metabolites were also definitively identified (Figure S2) and quantified using authentic standards (Section 2.3, Figure S3).

The Taverna workflows allow for integrated, automated and high-throughput annotation and putative metabolite identification from ESI LC-MS metabolomic data. The workflows consist of correlation analysis, metabolic feature annotation and metabolite annotation [37]. MarkerLynx XS data were exported into the Taverna workflows to be processed for metabolite identification (Section 3.6). The putative identities/annotations of mass ions selected from the PCA loading plot and VIP-plot were thus extracted from the Taverna workflow output file and are presented in Table 1 that lists 29 selected metabolites that are positively or negatively correlated with the treatment. The variation in intracellular metabolite profiles indicated by inspection of BPI chromatograms (Figure 1) and explained by PCA (Figure 2) is therefore a reflection of the cellular reprogramming occurring in *C. asiatica* cells in response to the MeJa treatment. 

Among plant secondary metabolites, terpenoids constitute the largest and structurally diverse family of natural products [38,39,40,41,42]. This ‘terpenome’ plays diverse functional roles in plants as photosynthetic pigments (e.g., phytol, carotenoids), hormones (e.g., gibberellins, abscisic acid), mediators of polysaccharide assembly (e.g., polyprenyl phosphates), electron carriers (e.g., ubiquinone, plastoquinone), and structural components of membranes (phytosterols). Furthermore, many specific terpenoids in the C_10_, C_15_ and C_20_ families serve in communication and defence responses [43].

The results obtained in this study showed that MeJa is able to trigger differential changes in the metabolome of *C. asiatica* cells, leading to a change in the biosynthesis of a broad range of secondary metabolites. A study of the effect of MeJa on production of centellosides *vs.* phytosterols [5] suggested that a decrease in free phytosterols was due to a competition between the two pathways, conducting the carbon flow through the centellosides pathway. Our data, similarly, do not indicate the presence of detectable levels of phytosterols in the MeJa-treated cells. Instead, all four of the targeted centellosides were found in the treated cells at statistically significant levels in order to be identified as signatory bio-markers. From Table 1 it appears that metabolites derived from the terpenoid pathway are positively correlated with the cellular response to MeJa and, interestingly, that the flavonoids exhibit a negative correlation. This might be due to the antagonistic cross-talk between the jasmonates and salicylic acid as signalling molecules involved in stress responses [44], as the flavonoids are derived from the phenylpropanoid pathway that is responsive to salicylic acid. The MeJa treatment of the *C. asiatica* cells therefore not only led to the reprogramming of the terpenoid pathway, but also the flavonoid pathway.

### 2.3. MeJa Treatment of *C. asiatica* Cells Enhances the Production of Triterpenoids

The quantitative analysis of the MeJa effect on secondary metabolism of *C. asiatica* cell suspensions was carried out by quantifying the targeted triterpenoids (asiaticoside, madecassoside, asiatic acid and madecassic acid) in the ethanolic extracts from the control and treated cell suspensions. Authentic standards of the four targeted compounds were used and the UHPLC-MS/MS (single ion reaction monitoring mode) analyses were performed for sensitive and specific quantitation of the targeted triterpenoids. QuanLynx™ 4.1 tool of the MassLynx XS^TM^ software was used for automated quantification (Figure S3) and the results are shown in Table 2.

**Table 2:** Quantification of the targeted pentacyclic triterpenoids (centellosides) in 0.2 mM MeJa-treated cell suspensions of *C. asiatica.*

Triterpenoid	Rt (min)	*m/z*	Time point (day)	Concentration (μg/g wet weight) Control Treated	
Madecassoside	9.49	973.51	2	0.388 ± 0.022	0.416 ± 0.024
			4	0.426 ± 0.044	10.580 ± 0.634
			6	0.424 ± 0.008	9.869 ± 9.235
Asiaticoside	10.05	957.51	2	0.669 ± 0.051	0.480 ± 0.146
			4	0.542 ± 0.093	20.934 ± 6.089
			6	0.727 ± 0.087	20.819 ± 5.685
Madecassic acid	12.41	503.33	2	0.515 ± 0.459	0.472 ± 0.489
			4	0.473 ± 0.450	8.065 ± 9.435
			6	0.539 ± 0.514	5.854 ± 6.381
Asiatic acid	13.47	487.34	2	0.258 ± 0.019	0.266 ± 0.011
			4	0.205 ± 0.010	4.859 ± 0.342
			6	0.317 ± 0.047	2.595 ± 0.096

The amount of accumulated targeted centelloids increased by the fourth day of 0.2 mM MeJa treatment, with an approximate 20–40 times increase in comparison to the non-treated controls (Table 2). The main pentacyclic triterpenoid was asiaticoside followed by madecassoside, with lower quantities of asiatic and madecassic acid. This corresponds to the findings of Mangas *et al*. [5] who reported a similar centelloid pattern in the aerial part and roots of the *C. asiatica* plant. This, however, contrasts to Bonfill *et al.* [15], who found the main compound in elicited cell suspensions to be madecassoside followed by asiaticoside. The triterpenoid pattern in *Centella* has been found to differ according to the species and culture region [4,45,46] although both asiaticoside and madecassoside appear to be prominent compounds in all cases [4,5].

## 3. Experimental

### 3.1. Preparation and Elicitation of Cell Cultures

Cell suspensions were grown and maintained as previously described [23]. For the experiments, 10 mL of the homogenous cell suspension was subcultured with 40 mL fresh culture medium in 100 mL Erlenmeyer flasks and grown for 10 days. For concentration studies, cell suspensions were treated by the application of MeJa (Sigma-Aldrich, Munich, Germany) to obtain final concentrations of 0.05–0.3 mM, with 0.2 mM found to be optimal and which is consistent with other studies [20,21,22]. The time study was done for 2, 4 and 6 days. Treatments with MeJa commenced at day 10 of culture growth, when the cells had attained the stationary phase of the growth curve [23]. Non-treated *C. asiatica* cell suspensions were used as negative controls for the respective time points. All results are based on three biological repeats.

### 3.2. Cell Viability Assessment

To verify that the cells were viable after treatment with MeJa, the Alamar blue™ assay [47] was performed. 200 µL of the suspensions were sampled and the medium removed by filtration. These cells were incubated with 180 µL 0.05 M sodium phosphate buffer, pH 7.4, and 20 µL Alamar blue^®^ (AbD Serotec, Kidlington, UK) for 1 h with gentle agitation in the dark. The cells were then sonified (50% power for 30 s) and centrifuged at 15,871 *×g* in a microcentrifuge for 10 min. The supernatant (180 µL) was transferred into a 24 (6 × 4) well microtiter plate and readings were taken at an excitation wavelength of 540 nm and emission wavelength of 620 nm on a Fluoroscan Ascent fluorimeter (AEC-Amersham, Johannesburg, South Africa). A blank sample was prepared with 20 µL Alamar blue™ and 180 µL 0.05 M sodium phosphate buffer, pH 7.4. After 4 days of 0.2 mM MeJa treatment (a total of 13 d in batch culture) the cells still maintained a viability of >80%.

### 3.3. Metabolite Extraction

Ethanolic extracts were prepared as previously described [23,24]. Briefly, after the required growth period was achieved, the medium was removed by filtration, and 3 g of cells were added to absolute ethanol (Laboratory and Control Monitoring, Johannesburg, South Africa) in a 1:3 (w/v) ratio. These were left overnight on an orbital shaker and the suspensions were centrifuged at 2,200 *×g* for 20 min. The supernatants were collected and vacuum dried to remove the solvent. The remaining residue was reconstituted into 2 mL absolute ethanol and filtered through a 0.22 µm filter into pre-labelled UHPLC vials fitted with slitted caps (Separations, Johannesburg, South Africa). 

### 3.4. TLC Analysis of Ethanolic Extracts

TLC analysis of the crude ethanolic extracts allowed for the screening and partial fractionation and characterization of the extracted *C. asiatica* metabolites. The concentrated crude cell suspensions extracts were reconstituted in a minimal amount of ethanol and applied to Silica gel 60 F_254_ TLC plates (20 × 20 cm, Merck, Darmstadt, Germany). These were developed in a chloroform, glacial acetic acid, methanol and dH_2_O (60:32:12:8 v/v/v/v) develop­ing solution. The plates were visualized under UV (366 and 254 nm) light to assess for any fluorescent compounds followed by detection of the triterpenoids by spraying with anisaldehyde-sulphuric acid (AS) reagent [24].

### 3.5. Ultra-High Performance Liquid Chromatography–High Definition Mass Spectrometry (UHPLC-HDMS) Analysis

LC-MS analyses were performed on a Waters Acquity UHPLC system coupled in tandem to a Waters photodiode array (PDA) detector and a SYNAPT G1 HDMS QTOF mass spectrometer (Waters Corporation, Milford, MA, USA). Chromatographic separation of the extracts was achieved on a Waters Acquity UHPLC column (BEH C_18_ 150 mm × 2.1 mm, 1.7 μm) thermostatted at 60 °C and gradient elution was performed. A binary solvent was utilized, consisting of water with 0.1% formic acid (eluent A) and acetonitrile (Romil Pure Chemistry, Cambridge, UK) (eluent B). The initial conditions were 95% A at a flow rate of 0.4 mL/min and kept constant for 1 min. A gradient elution was introduced to change the chromatographic conditions to 10% A at 16 min. The conditions were kept constant for 2 min to flush the analytical column, followed by restoring the column to the initial conditions at 18 min followed by equilibrium for 2 min. The total run time was 20 min and the injection volume was 10 μL. All measurements were done in triplicate to account for any analytic variability. The PDA scan was from 200 to 500 nm (1.2 nm resolution) with a sampling rate of 20 spectra per second.

The SYNAPT G1 HDMS was used in V-optics and operated in the electrospray ionisation (ESI) mode to detect the compounds of interest. To obtain typical mass accuracies between 1 and 3 mDa, leucine enkephalin (50 pg/mL) was used as a reference calibrant. Both ESI positive and negative mode for MS analysis was initially investigated, but following comparative evaluation, all subsequent analyses were performed in ESI^-^ mode with a capillary voltage of 3 kV and the sampling cone voltage at 60 V and the extraction cone at 4 V. The scan time was 0.1 s covering the 100 to 1,000 Da range. The source and desolvation temperature was set at 120 °C and 450 °C respectively. Nitrogen gas was used as the nebulisation gas at a flow rate of 800 L/h. The software used to control the hyphenated system and perform all data manipulation was MassLynx 4.1 (Waters Corporation, Manchester, UK).

Samples were analysed in three biological repeats for treated and control conditions, each with three analytical replicates (n = 9). In addition, each analytical replicate was analysed in triplicate to include technical repeats.

For quantification of the targeted compounds (asiatic acid, madecassic acid, asiaticoside and madecassoside) authentic standards of these four compounds (Extrasynthase, Lyon, France) were used and a concentration range of 0–1,000 ng/mL was prepared for calibration curves. The UHPLC-MS/MS (single ion reaction monitoring mode) analyses were performed for sensitive and specific quantitation of the targeted triterpenoids. For these analyses, the chromatography was performed as described above and mass spectrometry was operated in MS/MS mode with a set mass corresponding to *m/z* of each targeted compound (487.3 Da for asiatic acid, 503.3 Da for madecassic acid, 957.5 Da for asiaticoside and 973.5 Da for madecassoside) in the mass range of 100–1,000 Da. The scan time was 0.1 s, with interscan time of 0.02 s and time range of 0–20 min. The other MS parameters were set as described above.

### 3.6. Data Analysis

PCA modelling was used for the analysis of the UPLC-ESI-MS data. The ESI^-^ raw data were extracted and analysed using MassLynx XSsoftware (Waters Corporation, Manchester, UK). Software parameters were set to analyse the 6–17 min Rt range of the chromatogram, mass range 200–1,000 Da, mass tolerance 0.05 Da, mass window 0.05 Da and a Rt window of 0.20 min. The MarkerLynx data matrix (of Rt-*m/z* variable pairs, with the *m/z* peak intensity for each sample), was exported to the SIMCA-P 13 (Umetrics, Umea, Sweden) software and *Pareto*-scaled for PCA modelling and VIP analysis. PCA scores and loading plots were used to explain variations in the samples [48]. 

Automated quantification was performed using QuanLynx4.1 tool of the MassLynx software. The same raw data were processed with QuanLynx and the targeted analytes were identified by the accurate mass of their respective [M−H]^−^ precursor ion and retention time. The chromatogram mass window was set to 0.02 Da and the retention time window was ±2.4 min. Peak area was selected as response type and mean smoothing method was applied. All standard curves had *r^2^* values of 0.99 (Figure S3).

For metabolite identification, the data matrix from MarkerLynx processing was exported to the Taverna workbench (www.taverna.org.uk) [36] for PUTMEDID_LCMS metabolite identification workflows. The Taverna workflows allow for integrated, automated and high-throughput annotation and putative metabolite identification from ESI LC-MS metabolomic data. The workflows consist of correlation analysis, metabolic feature annotation and metabolite [37]. The data matrix from MarkerLynx processing was firstly formatted to match the Taverna workbench requirements. Three main workflows formed the Taverna Metabolite ID procedure. In the workflow 1 (*List_CorrData*), all the parameters were set at default, ion mode selected as negative and Pearson correlation calculation was chosen as the correlation method. In the workflow 2 (*annotate_Massmatch*), mass tolerance was set to 5 ppm, retention time range set as 180–1,020 s (6–17 min) and all other parameters were set at default. These workflows allowed for grouping together ion peaks with similar features such as Rt, and annotating features with the type of *m/z* ion (molecular ion, isotope, adduct, others) believed to originate from the same compound. The elemental composition / molecular formula (MF) of each *m/z* ion is then automatically calculated. In workflow 3 (*matchMF-MF*) the calculated MF (from the output file from workflow 2) is automatically compared and matched to the MF from a pre-defined reference file of metabolites. The calculated MF can also be manually searched against freely available online databases such as Dictionary of Natural Products, Chemspider, PlantCYC, KEGG and METLIN.

## 4. Conclusions

Metabolic profiling and metabolomic techniques are reliable and well-studied investigative tools and the use thereof provides a new approach to investigate the effects of exogenous MeJa as a signalling molecule and elicitation agent on *C. asiatica* cell suspensions. The manipulation of the centelloid metabolic profiles of *C. asiatica* cell suspensions by external addition of MeJa was found to be feasible, with both quantitative and qualitative changes occurring. The combination of PCA with metabolic profiling clearly demonstrated the metabolic changes that occurred in the cells as a function of time through clustering of data values obtained at 2, 4 and 6 days. Asiaticoside and asiatic acid as well as madecassoside and madecassic acid were discriminatory biomarkers in the treated extracts, confirming the increase in their concentrations due to MeJa treatment. In addition, preliminary data also indicated a wider reprogramming of secondary metabolite pathways. MeJa therefore seems to target not only the pentacyclic triterpenoid pathway, but also other branches of the terpenoid metabolic tree in *C. asiatica*.

## Supplementary Materials

Supplementary materials can be accessed at: http://www.mdpi.com/1420-3049/18/4/4267/s1.
