# Supplementary material for: Metabolomic Analysis of Methyl Jasmonate-Induced Triterpenoid Production in the Medicinal Herb Centella asiatica (L.) Urban
Source: Molecules. 2013 Apr 11;18(4):4267–81. doi: 10.3390/molecules18044267 (PMC6270148; doi:10.3390/molecules18044267)

Supplementary Materials

Figure S1. The principal component analysis (PCA) of the UPLC-MS data (ESI negative) of all experiments: The ethanolic extracts of the *C. asiatica* cell suspensions treated with 0.2 mM MeJa and incubated for different time period (2, 4 and 6 day). A non-supervised modelling (PCA) of the data resulted in a 10-component model explaining 86.4% variation. The obtained model gave a very good fit to the data ($R^2X = 0.864$) with a reliable predictive accuracy ($Q^2 = 0.756$). The scores plot (below) was computed using the first two components (PC1 and PC2) which explain 44.5% of the variation. The plot clearly indicates the samples being differentially clustered in different treatment-defined groups. The non-treated samples (controls: open-triangle shapes) are significantly separated from the 0.2 mM MeJa-treated samples (open diamond shaped).

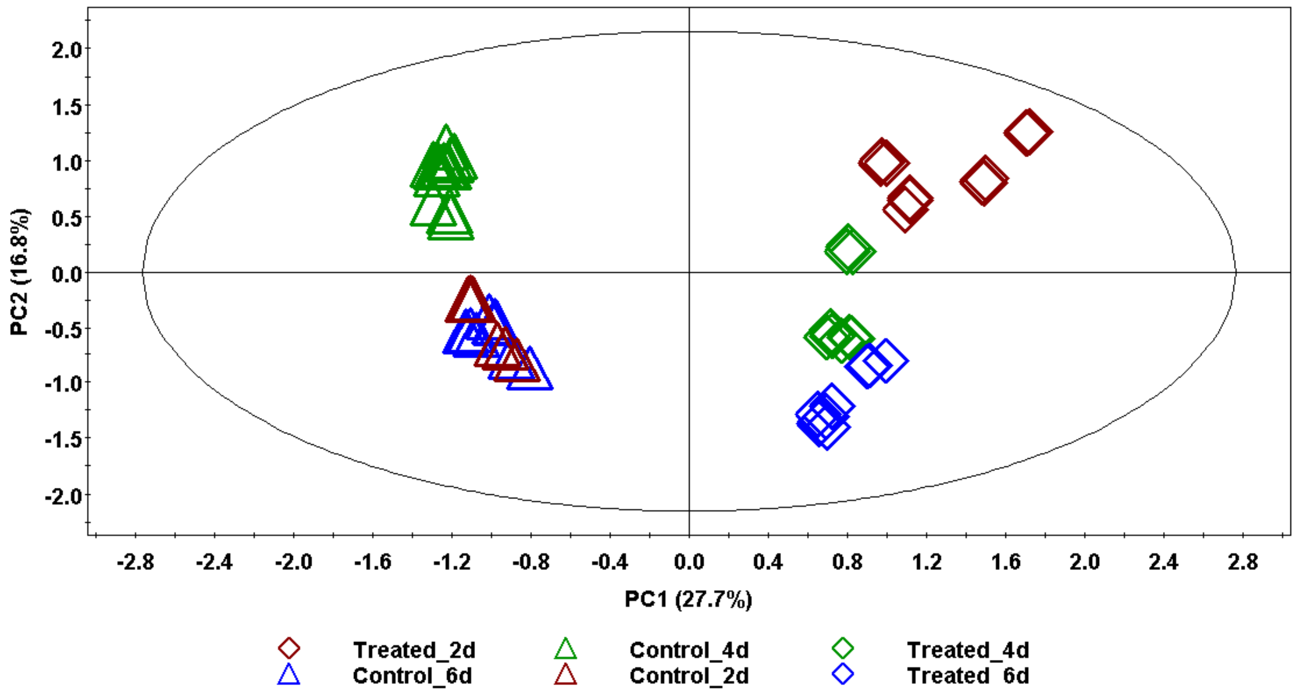

Figure S2. (**A**) UHPLC-MS (BPI) chromatogram of the four authentic standards (Extrasynthase, Switzerland) mixed together (Madecassoside, asiaticoside, madecassic acid and asiatic acid): The LC-MS analyses of the four standards provided two parameters (retention time, Rt and mass-to-charge ratio, *m/z*) that enabled the definitive identification of the pentacyclic triterpenoids in the samples; (**B**) UHPLC-MSMS (BPI) chromatograms of the four mass ions (*m/z* 973.50, 957.50, 503.33 and 487.34) that were definitively identified as madecassoside, asiaticoside, madecassic acid and asiatic acid, respectively.

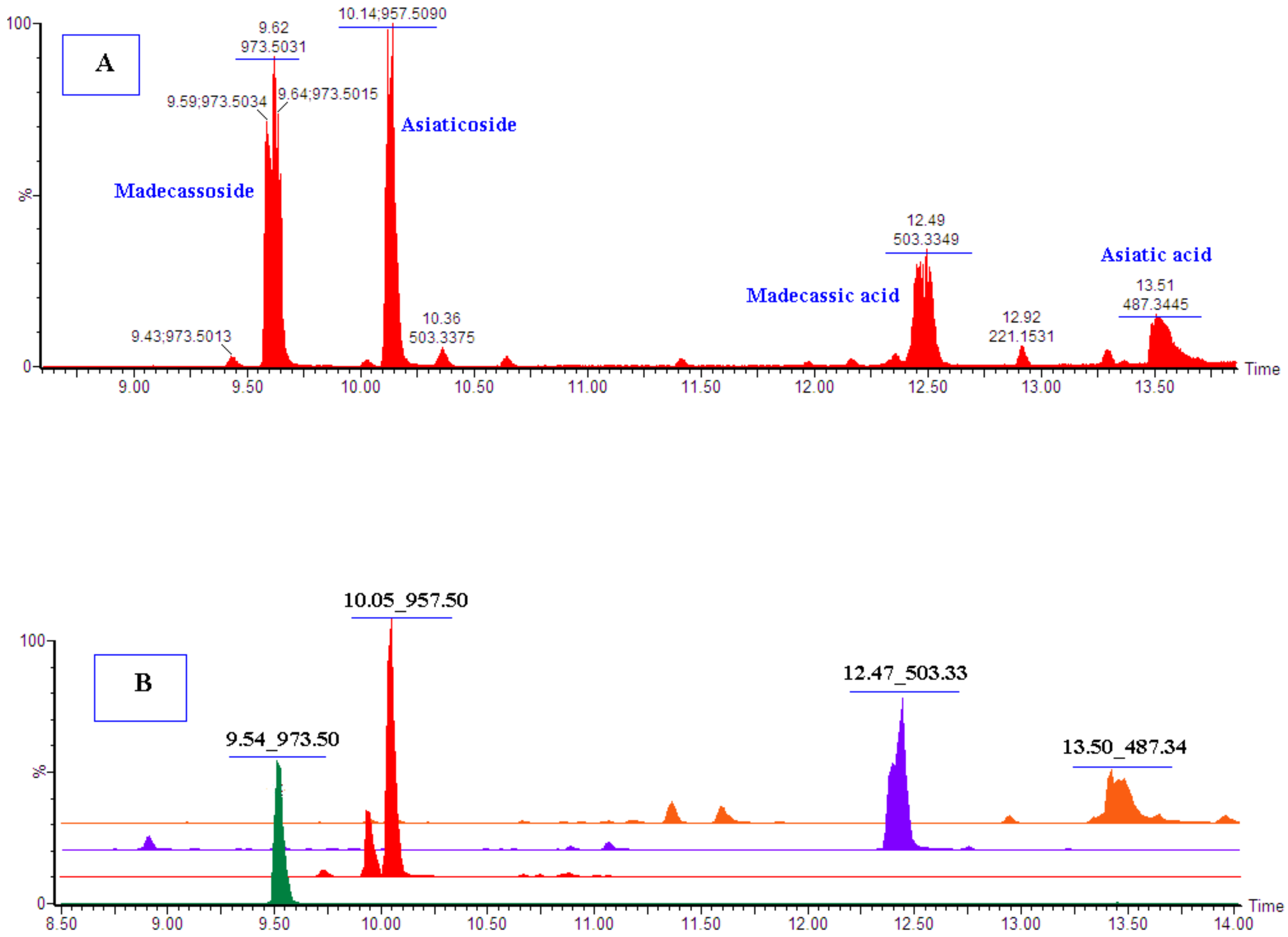

Figure S3. Calibration curves generated from QuanLynx-based quantification of (**A**) asiatic acid, asiaticoside and (**B**) madecassic acid and madecassoside. As indicated in the respective figures, the types of curves used were second order (for asiatic acid) and third order for the other three compounds (because of the non-linearity of ESI-MS). The obtained curves had r^2 = 0.99. The calibration curve equations are also indicated on the figures. The concentration values (of the four targeted compounds) obtained using these standard curves were then converted to µg/g wet weight (as reported in Table 2).

A

Compound name: Asiatic acid
Coefficient of Determination: R^2 = 0.999877
Calibration curve: 53.4355 * x^2 + 84.2592 * x + 0.13988
Response type: External Std, Area
Curve type: 2nd Order, Origin: Include, Weighting: Null, Axis trans: None

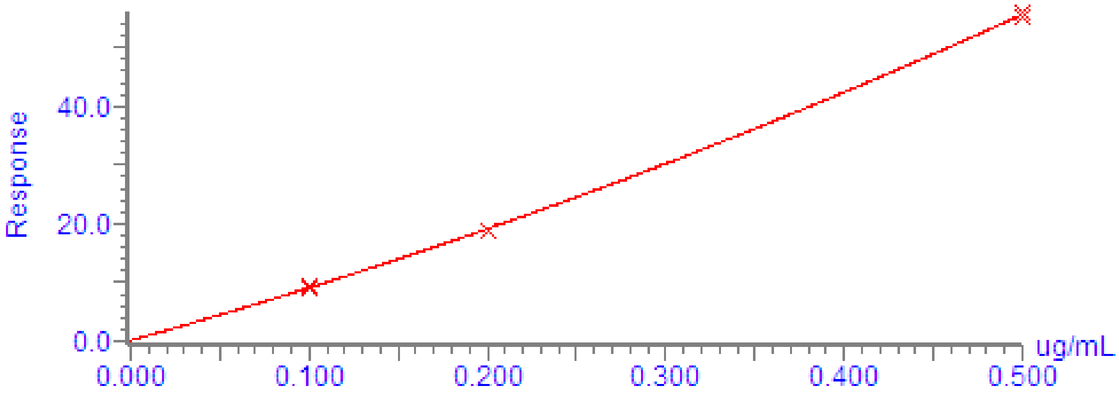

Compound name: Asiaticoside
Coefficient of Determination: R^2 = 0.999474
Calibration curve: -0.919468 * x^3 + 8.12152 * x^2 + 28.8832 * x
Response type: External Std, Area
Curve type: 3rd Order, Origin: Force, Weighting: Null, Axis trans: None

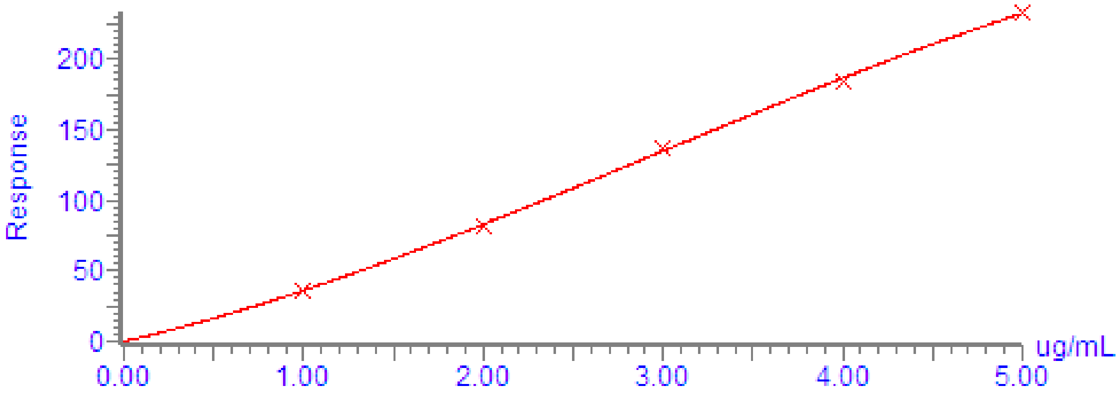

Figure S3. *Cont.*

B

Compound name: Madecassic acid
Coefficient of Determination: R^2 = 0.999527
Calibration curve: -37.0322 * x^3 + 72.0148 * x^2 + 0.293006 * x
Response type: External Std, Area
Curve type: 3rd Order, Origin: Force, Weighting: Null, Axis trans: None

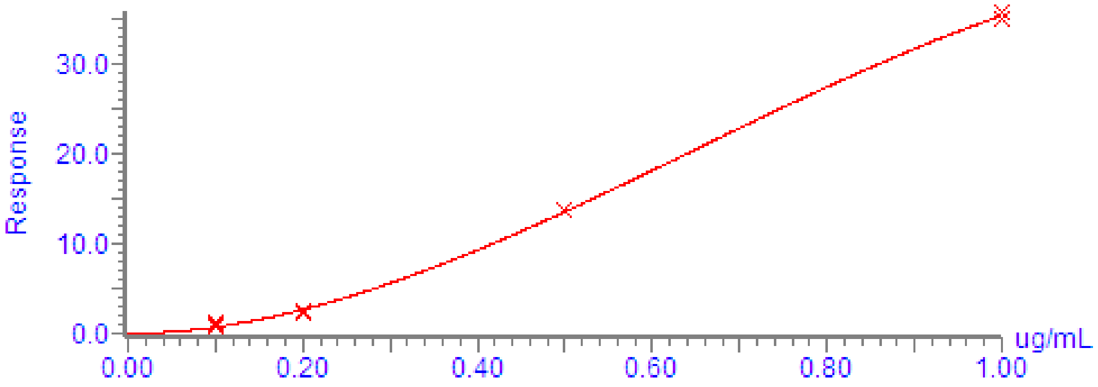

Compound name: Madecassoside
Coefficient of Determination: R^2 = 0.999849
Calibration curve: -39.6348 * x^3 + 69.3776 * x^2 + -0.389563 * x
Response type: External Std, Area
Curve type: 3rd Order, Origin: Force, Weighting: Null, Axis trans: None

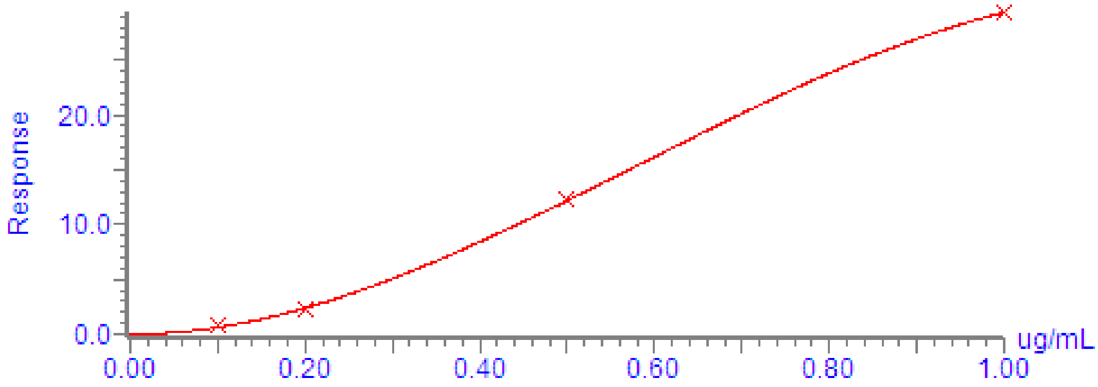

Supplement: Supplementary file 1 [file molecules-18-04267-s001.pdf]
